# Gram-negative bacterial infection randomized controlled trials (RCTs) in the 21st century: characteristics and evolution

**DOI:** 10.1093/jacamr/dlag122

**Published:** 2026-07-23

**Authors:** Fabian Patauner, Hyeri Seok, Navaneeth Narayanan, Jason M Pogue, Emanuele Durante-Mangoni, Keith S Kaye

**Affiliations:** Department of Medicine, Rutgers Robert Wood Johnson Medical School, New Brunswick, NJ 08901, USA; Department of Precision Medicine, University of Campania ‘L. Vanvitelli’, Naples, Italy; Department of Medicine, Rutgers Robert Wood Johnson Medical School, New Brunswick, NJ 08901, USA; Department of Medicine, Korea University Medicine, Seoul, South Korea; Department of Medicine, Rutgers Robert Wood Johnson Medical School, New Brunswick, NJ 08901, USA; Department of Pharmacy Practice and Administration, Rutgers Ernest Mario School of Pharmacy, Piscataway, NJ, USA; Department of Clinical Pharmacy, University of Michigan College of Pharmacy, Ann Arbor, MI, USA; Department of Precision Medicine, University of Campania ‘L. Vanvitelli’, Naples, Italy; AORN Ospedali dei Colli, Monaldi Hospital, Naples, Italy; Department of Medicine, Rutgers Robert Wood Johnson Medical School, New Brunswick, NJ 08901, USA

## Abstract

Since 2000, several randomized controlled trials (RCTs) addressing treatment of infections due to Gram-negative bacilli (GNB) have been published. We reviewed RCTs targeting GNB infections published between 2000 and 2025, focusing on methodology, generalizability of results and impact on patient care. Included were phase 2–4 RCTs enrolling adults with complicated intrabdominal infections (cIAI), hospital-acquired pneumonia/ventilator-associated pneumonia (HAP/VAP), complicated urinary tract infections (cUTI) and/or bloodstream infections (BSI). Sixty-eight studies were included. cIAIs and cUTIs were the most frequent infection syndromes studied [in 30 (44.1%) and 28 (41.2%) RCTs, respectively]. Forty-two (61.8%) studies used a double-blind design, and 30 (44.1%) entailed placebo administration. Non-inferiority was the most common pre-specified hypothesis tested (48 RCTs, 70.6%). Superiority was assessed in only four studies. Twenty-nine unique antibiotics were evaluated overall, with novel agents assessed in 43 studies (63.2%). Twelve RCTs targeted one or more specific pathogen, three of which (25.0%) addressed a single pathogen, *Acinetobacter baumannii* in two RCTs and *Escherichia coli* in one. All of the 12 studies focused on specific antimicrobial resistant pathogens: 3 (25.0%) on third-generation cephalosporin-resistant GNB and/or ESBL-producers and 9 (75.0%) on carbapenem-resistant pathogens.

In summary, many high-quality RCTs of GNB infection treatment have been published in the 21st century. Results of many of these studies were influenced by methodological limitations and low rates of inclusion of multi-drug-resistant GNB, reducing generalizability to real-world clinical practice. Increased funding opportunities and regulatory modifications would facilitate conduct of real-world RCTs of GNB.

## Introduction

Gram-negative bacilli (GNB) are a frequent cause of serious infection. According to a recent ECDC report, the two most frequently isolated GNB are *Escherichia coli* (41%) and *Klebsiella pneumoniae* (15%).^1^ GNB are not only responsible for most infections that occur during hospital admissions but are also frequent causes of community-acquired infections. Indeed, one-third of all GNB bloodstream infections diagnosed in the hospital are community-acquired.^2^

The 21st century has seen the emergence and global spread of several GNB strains that express a variety of resistance determinants. These multi-drug-resistant (MDR) pathogens^3^ have been associated with increased morbidity and mortality compared to susceptible phenotypes.^4,5^ In the 2024 WHO Bacterial Priority Pathogens List, GNB maintained their high-priority status with regards to need for research and development of therapeutic agents.^6^ Among these high-priority GNB, carbapenem-resistant *Enterobacterales* together with carbapenem-resistant *Acinetobacter baumannii* (CRAB) and third-generation cephalosporin-resistant *Enterobacterales* received the highest scores.^6^

The 21st century has also witnessed the development of several novel antimicrobials, some of which were designed specifically to address the emerging MDR GNB threat.^7^ Randomized controlled trials (RCTs) are considered the gold-standard study design to evaluate the efficacy of antimicrobials. Data from RCTs are required for novel antimicrobials to be considered for FDA and EMA approval. Unfortunately, RCTs often fail to address ‘real-world’ clinical questions and scenarios, precluding the generalizability of results to certain populations such as severely ill or immunocompromised patients. In addition, in some instances, the same key MDR pathogens targeted by a novel agent are excluded from pivotal phase III RCTs, and as a result, MDR GNB often represent a very small share of included pathogens in these trials.

Given the sharp increase in the number of GNB RCTs performed after 2000 and the challenges in conducting clinically relevant, real-world trials involving MDR GNB, we felt it important to review RCTs targeting GNB that were published in the 21st century. The aims of this study were to critically analyse the study design, inclusion and exclusion criteria, outcomes, generalizability of the results and impact on real-world clinical care of these RCTs.

## Methods

### Literature search

We performed a literature review through PubMed database searching for articles published between 1 January 2000 and 8 August 2025. The following key words were used to select articles: Gram-negative, resistant, BL-BLI, carbapenem, cephalosporin, cefiderocol, meropenem, imipenem, tebipenem, ceftazidime, avibactam, ceftolozane, sulbactam, durlobactam, relebactam, taniborbactam, colistin, polymyxin, tigecycline, eravacycline, fosfomycin and plazomicin.

### Study selection, data extraction and quality assessment

We included phase 2, 3 and 4 RCTs that included adult patients with at least one of the following clinical conditions: complicated intrabdominal infections (cIAI), hospital-acquired pneumonia/ventilator-associated pneumonia (HAP/VAP), complicated urinary tract infections (cUTI) or bloodstream infections (BSI) treated with a systemic antibiotic. We excluded studies that: (i) primarily targeted patients with cystic fibrosis; (ii) had fewer than 50 patients included in each arm of the primary outcome analysis population; (iii) studied uncomplicated UTI; (iv) primarily focused on investigating different doses or treatment intervals of a single drug and (v) for which the full article was not accessible. Six clinical trials of newly developed antibiotics^8–13^ that were used for regulatory submission were included despite having <50 patients in each treatment arm.

From each included article, the following data were collected: general characteristics of the study (e.g. authors, year of publication, geographical areas and funding source), study design/methodology (e.g. the use of double-blinding, placebo control and non-inferiority assessment), class, administration route and dosages of investigational and control drugs, major inclusion and exclusion criteria, the definition and timing of assessment of the primary outcome and microbiological data reported (including frequently detected GNB, the presence of MDR strains and underlying resistance mechanisms of MDR pathogens). For each trial, the three countries with the highest rates of enrolment were listed, when this information was available. A study was classified as industry funded when the primary sponsor was a pharmaceutical company.

### Statistical analyses

Summary data were presented as numbers and percentages for categorical variables and medians with IQRs for continuous variables. Linear regression was performed to analyse for the presence of trends over time. All figures and analyses were performed using Excel, Microsoft 365 v.2511.

## Results

**Figure 1.**
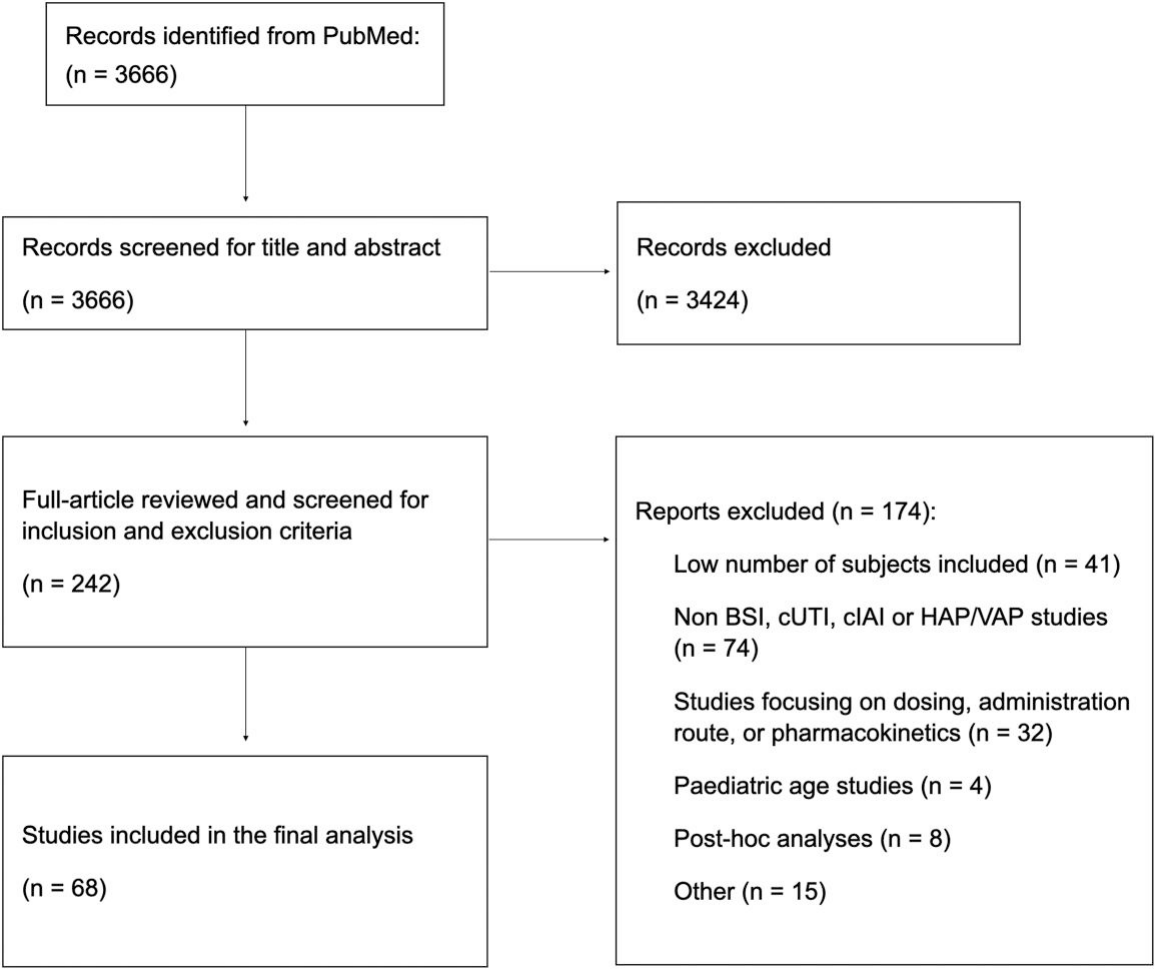
Flow diagram of the study. Sequential phases of literature search and article selection screening process are shown.

In total, 3666 articles were retrieved and screened for inclusion and exclusion criteria. After applying these criteria, 68 articles were included in the final analysis. A flow diagram detailing the reasons for exclusion of studies is shown in Figure 1. Characteristics of the 68 studies selected are presented in Table 1.

**Table 1.** Characteristics of RCTs by clinical syndromes

Trial	Intervention (duration)	Control (duration)	Study design^a^	Major enrolment countries	Primary analysis population: intervention group sample size; control group sample size	Primary outcome (Time frame); Results intervention versus control Primary outcome results	Major pathogens	Resistance type
**Multiple clinical syndromes**								
ASSEMBLE: Daikos *et al.*, 2025^12^ (BSI, HAP/VAP, cUTI, cIAI)	Aztreonam/Avibactam 2 gr IV q6h ± Metronidazole (Mean 9.5 days, Range 1–15)	BAT (Median 4.5 days, Range 4–5)	NA	NA^b^	micro-ITT: ATM-AVI 12, BAT 3	Clinical cure (Day 25–31); 41.7% versus 0% The study was early terminated	*K. pneumoniae*, *E. coli*, *S. maltophilia*	MBL-GN
REVISIT: Carmeli *et al.*, 2025^14^ (HAP/VAP, cIAI)	Aztreonam/Avibactam 2 g IV q6h (Mean 8.5 days, SD 3.5)	Meropenem 1 g IV q8h (Mean 8.9 days, SD 3.2)	NA	China, Spain, India	ITT: ATM-AVI 282; MEM 140 CE: ATM-AVI 213; MEM 105	Clinical cure (Day 25–31); ITT: 68.4% versus 65.7%, RD: 2.7 (95% CI −6.6 to 12.4) CE: 77.0% versus 74.3%, RD: 2.7 (95% CI −7.0 to 13.2)	*E. coli*, *K. pneumoniae*, *P. aeruginosa*	3GCR-E and CR-E^c^
ATTACK: Kaye *et al.*, 2023^15^ (BSI, HAP/VAP, cUTI, cIAI)	Sulbactam/Durlobactam 2 g IV q6h + Imipenem/Cilastatin 2 g q6h (Range 7–14 days)	Colistin 2.5 mg/kg IV every q12h + Imipenem/Cilastatin 2 g q6h (Range 7–14 days)	Non-inferiority	NA^b^	micro-MITT: SUL-DUR 63; CST 62	All-cause mortality (Day 28); 19% versus 32%, RD: −13.2 (95% CI −30.0 to 3.5) Non-inferiority met	*A. baumannii*	CRAB
OVERCOME: Kaye *et al.*, 2022^16,d,e,f^ (HAP, BSI)	Meropenem 1 g IV q8h + Colistin 1.67 mg/kg IV q8h (Range 7–14 days)	Colistin 1.67 mg/kg IV q8h + Placebo (Range 7–14 days)	Superiority	NA^b^	modified-ITT: MEM + CST 210; CST 213	All-cause mortality (Day 28); 37% versus 43%, RD: 6.5 (95% CI −2.8 to 15.8), *P* = 0.17 Superiority not met	*A. baumannii*, *CR-E*, *P. aeruginosa*	CRAB, CR-E, CR-P
CREDIBLE-CR: Bassetti *et al.*, 2021^9^ (BSI, HAP/VAP, cUTI)	Cefiderocol 2 g IV q8h (Median 11.0 days, IQR 8.0–14.0)	BAT (Median 13.0 days, IQR 10.0–15.0)	NA	NA^b^	micro-ITT: FDC 80; BAT 38	Clinical cure (BSI-HAP/VAP, Day 5–9 after EOT); BSI: 43% versus 43%, HAP/VAP: 50% versus 53% Microbiological cure (cUTI, Day 5–9 after EOT); 53% versus 20%	*A. baumannii*, *K. pneumoniae*, *P. aeruginosa*	CR-E, CRAB
CARE: McKinnell *et al.*, 2019^11^ (BSI, HAP/VAP)	Plazomicin 15 mg/kg q24h + Meropenem or Tigecycline IV	Colistin 5 mg/kg/day IV + Meropenem or Tigecycline IV	NA	Greece, Brasil^b^	micro-MITT: PLA 17; COL 20	Composite of death from any cause at 28 days or clinically significant disease-related complications (Day 28); 24% versus 50%, RD: −26 (95% CI −55 to 6) The study was early terminated	*K. pneumoniae*, *E. aerogenes*	CR-E
RESTORE-IMI 1: Motsch *et al.*, 2019^13,e,f^ (HAP/VAP, cUTI, cIAI)	Imipenem/Relebactam 500/250 mg IV q6h + Placebo (Mean 11.4 days, Range 2–18)	Colistin 150 mg IV q12h + Placebo (Mean 10.8 days, Range 2–20)	NA	Ukraine, Germany, Turkey	micro-MITT: I-R 21, COL 10	Overall response; 71.4% versus 70.0%, RD: −7.3 (95% CI −27.5 to 21.4) cUTI: clinical and microbiological response (Day 5–9 after EOT); 72.7% versus 100%, RD: −27.3 (95% CI −52.8 to 12.8) HAP/VAP: all-cause mortality (Day 28); 87.5% versus 66.7%, RD: 20.8 cIAI: clinical cure (Day 28); 0% versus 0%	*P. aeruginosa*, *K. pneumoniae*	CR-P, CR-E
AIDA: Paul *et al.*, 2018^17,d^ (BSI, HAP/VAP, cUTI)	Meropenem 2 g IV q8h + Colistin 4.5 MIU IV q12h	Colistin 4.5 MIU IV q12h	Superiority	Israel, Greece, Italy	ITT: MEM + CST 208; CST 198	Clinical failure (Day 14); 73% versus 79%, RD: −5.7 (95% CI −13.9 to 2.4) Superiority not met	*A. baumannii*, *K. pneumoniae*, *P. aeruginosa*	CR-E, CRAB
TANGO II: Wunderink *et al.*, 2018^8^ (BSI, HAP/VAP, cUTI, cIAI)	Meropenem/Vaborbactam 4 g IV q8h	BAT	NA	NA^b^	mCRE-MITT: MEV 32; BAT 15	Overall success (Day 5–9 after EOT); 59.4% versus 26.7%, RD: 32.7 (95% CI 4.6 to 60.8), *P* = 0.02 cUTI: overall success (Day 5–9 after EOT); 33.3% versus 50.0% HABP/VABP and BSI: all-cause mortality (Day 5–9 after EOT); 22.2% versus 44.4% cIAI: clinical cure (Day 5–9 after EOT); 100% versus 0%	*K. pneumoniae*, *E. coli*, *E. cloacae*	CR-E
REPRISE: Carmeli *et al.*, 2016^18^ (cUTI, cIAI)	Ceftazidime/Avibactam 2.5 g IV q8h (cUTI, Median 10 days, range 2–21, cIAI Median 10.5 days, Range 6–21)	BAT (cUTI, median 10 days, Range 2–21, cIAI median 12.0 days, Range 4–23)	NA	Russia, Bulgaria, Romania, Ukraine	micro-MITT: CZA 154; BAT 148	Clinical cure (Day 7–10 after EOT); 91% versus 91%	*E. coli*, *K. pneumoniae*, *P. aeruginosa*	3GCR-E
Durante-Mangoni *et al.*, 2013^19,d^ (BSI, HAP/VAP, cUTI, cIAI)	Rifampicin 600 mg IV q12h + Colistin 2 MU IV q8h (Median 12.5 days, IQR 8–17)	Colistin 2 MU IV q8h (Median 12.5 days, IQR 8–17)	Superiority	Italy	ITT: RIF + CST 104; CST 105	All-cause mortality (Day 30); 43.3% versus 42.9%, *P* = 0.95 Superiority not met	*A. baumannii*	CRAB
**Bloodstream infection**								
MERINO: Harris *et al.*, 2018^20,d^	Piperacillin/Tazobactam 4.5 g IV q6h (Median 6 days, IQR 5–10)	Meropenem 1 g IV q8h (Median 6 days, IQR 5–9)	Non-inferiority	Singapore, Australia, Turkey	Any correctly randomized patient receiving at least one dose of allocated drug: TZP 187; MEM 191	All-cause mortality (Day 30); 12.3% versus 3.7%, RD: 8.6 (one-sided 97.5% CI ≤ 14.5%), *P* = 0.90 Non-inferiority not met	*E. coli*, *K. pneumoniae*	3GCR-E^c^
**Hospital-acquired pneumonia/Ventilator-associated pneumonia (HAP/VAP)**								
Li *et al.*, 2025^21,e^	Imipenem/Relebactam 750 mg IV q6h	Piperacillin/Tazobactam 4.5 g IV q6h	Non-inferiority	China, Ukraine, Russia	modified-ITT: I-R 134; TZP 136	All-cause mortality (Day 28); 11.2% versus 5.9%, RD: 5.2 (95% CI −1.5 to 12.4), *P* = 0.02 Non-inferiority met	*K. pneumoniae*, *A. baumannii*, *P. aeruginosa*	3GCR-E, CR-E, CRAB
APEKS-NP: Wunderink *et al.*, 2021^22^	Cefiderocol 2 g IV q8h (Mean 10.4 days, SD 4.1)	Meropenem 2 g IV q8h (Mean 10.1 days, SD 4)	Non-inferiority	Asia, Europe, USA	modified-ITT: FDC 145; MEM 147	All-cause mortality (Day 14); 12.4% versus 11.6%, RD: 0.8 (95% CI −6.6 to 8.2), *P* < 0.01 Non-inferiority met	*K. pneumoniae*, *P. aeruginosa*, *A. baumannii*	3GCR-E, CR-E, CRAB^c^
RESTORE-IMI 2: Titov *et al.*, 2021^23,e^	Imipenem/Relebactam 750 mg IV q6h (Mean 8.7 days)	Piperacillin/Tazobactam 4.5 g IV q6h (Mean 8.3 days)	Non-inferiority	Ukraine, Brazil, Japan	modified-ITT: I-R 264; TZP 267	All-cause mortality (Day 28); 15.9% versus 21.3%, RD: −5.3 (95% CI −11.9 to 1.2), *P* < 0.01 Non-inferiority met	*K. pneumoniae*, *A. baumannii*, *P. aeruginosa*	
Magic Bullet: Cisneros *et al.*, 2019^24,d^	Colistin 3 MIU IV q8h + Levofloxacin 500 mg IV q12h (Median 9 days, IQR 5–14)	Meropenem 2 g IV q8h + Levofloxacin 500 mg IV q12h (Median 7.9 days, IQR 5–11)	Non-inferiority	Spain, Greece, Italy	micro-MITT: CST 82; MEM 75	All-cause mortality (Day 28); 23.2% versus 25.3%, RD: −2.2 (95% CI­ −15.6 to 11.3) Non-inferiority not met The study was interrupted prematurely	*A. baumannii*, *P. aeruginosa*, *K. pneumoniae*	CR-E, CRAB
ASPECT-NP: Kollef, *et al.*, 2019^25,e,f^	Ceftolozane/Tazobactam 3 g IV q8h (Median 7.7 days, IQR 7.3–9.7) ± Placebo	Meropenem 1 g IV q8h (Median 7.7 days, IQR 7.5–10.7) ± Placebo	Non-inferiority	Russia, Czech Republic, Ukraine	ITT: C-T 362; MEM 364	All-cause mortality (Day 28); 24% versus 25.3%, RD: 1.1 (95% CI −5.1 to 7.4) Non-inferiority met	*K. pneumoniae*, *P. aeruginosa*, *E.coli*	CR-E, CR- P
REPROVE: Torres *et al.*, 2018^26,e,f^	Ceftazidime/Avibactam 2.5 g IV q8h + Placebo	Meropenem 1 g IV q8h + Placebo	Non-inferiority	China, Czech Republic, Brazil	cMITT: CZA 356; MEM 370 CE: CZA 257; MEM 270	Clinical cure (Day 21–25); cMITT: 68.8% versus 73.0%, RD: −4.2 (95% CI −10.8 to 2.5), *P* < 0.01 CE: 77.4% versus 78.1%, RD: −0.7 (95% CI −7.9 to 6.4), *P* < 0.01 Non-inferiority met for all the primary analyses	*K. pneumoniae*, *P. aeruginosa*, *E. cloacae*	CR-E, CR-P
Freire *et al.*, 2010^27,e,f^	Tigecycline 50 mg IV q12 ± Placebo (Median 10 days)	Imipenem/Cilastatin 0.5 to 1 g IV q8h ± Placebo (Median 10 days)	Non-inferiority	NA^b^	CE: TGC 268; IPM 243 cMITT: TGC 440; IPM 429	Clinical response (Day 10–21 after EOT); CE: 67.9% versus 78.2%, RD: −10.4 (95% CI −17.8 to −3.0), *P* = 0.12 Non-inferiority not met cMITT: 62.7% versus 67.6%, RD: −4.8 (95% CI −11.0 to 1.3), *P* < 0.01 Non-inferiority met	*A. baumannii*, *K. pneumoniae*, *E. coli*	
Chastre *et al.*, 2008^28^	Doripenem 500 mg IV q8h (Mean 8.6 days)	Imipenem/Cilastatin 0.5 g IV q6h or 1 g IV q8h (Mean 9.0 days)	Non-inferiority	NA^b^	cMITT: DOR 249; IPM 252 CE: DOR 126; IPM 122	Clinical cure (Day 7–14 after EOT); cMITT: 59.0% versus 57.8%, RD: 1.2 (95% CI −7.9 to 10.3) CE: 68.3% versus 64.8%, RD: 3.5 (95% CI −9.1 to 16.1) Non-inferiority met for all the primary analyses	*P. aeruginosa*, *E. coli*, *K. pneumoniae*	CRAB, CR-*P*, *S. maltophilia*
Heyland *et al.*, 2008^29^	Ciprofloxacin 400 mg IV q12h + Meropenem 1 g IVq8h (Median 3 days, IQR 2–5)	Meropenem 1 g IV q8h (Median 3 days, IQR 2–5)	Superiority	Canada, USA	ITT: CIP + MEM 369; MEM 370	All-cause mortality (Day 28); RR: 1.05 (95% CI 0.78 to 1.42), *P* = 0.74 Superiority not met	*H. influenzae*, *Enterobacter spp.*, *Klebsiella spp.*	MDRO^i^
Schmitt *et al.*, 2006^30,e^	Piperacillin/Tazobactam 4.5 g IV q6h (Mean 8.7 days, SD 3.1)	Imipenem/Cilastatin 2 g IV q8h (Mean 9.0 days, SD 3.1)	NA	Germany, Czech Republic, Hungary	ITT: TZP 107; IPM 110	Clinical response (Day 3 after EOT); 66% versus 70% The study was early terminated	*Enterobacterales*, *P. aeruginosa*	c
Joshi *et al.*, 2006^31,e^	Piperacillin/Tazobactam 4.5 g IV q6h (Mean 8.7 days, SD 3.1)	Imipenem/Cilastatin 1 g IV q8h	NA	USA, Canada	EE: TZP 98; IPM 99	Clinical cure (Day 14 after EOT); 68.4% versus 60.6%, RD: 7.8 (95% CI −6.6 to 22.1), *P* = 0.26	*P. aeruginosa*, *K. pneumoniae*, *E. cloacae*	
Zanetti *et al.*, 2003^32^	Cefepime 2 g IV q8h	Imipenem/Cilastatin 0.5 g IV q6h	Non-inferiority	NA^b^	PP: FEP 108; IPM 101	Clinical response (EOT); 70% versus 74%, RD: −4 (95% CI −16 to 8) Non-inferiority not met	*P. aeruginosa*, *K. pneumoniae*, *A. baumannii*	3GCR-E^c^
West *et al.*, 2003^33^	Levofloxacin 750 mg IV q24h (Mean 8.2 days)	Imipenem/Cilastatin 0.5–1 g IV q6–8h (Mean 9.1 days)	Non-inferiority	USA	CE-ME: LVX 93; IPM 94	Clinical response (Day 3–15); 58.1% versus 60.6%, RD: 2.5 (95% CI −12.0 to 17.2) Non-inferiority met	*P. aeruginosa*, *E. coli*, *K. pneumoniae*	c
**Complicated intra-abdominal infection (cIAI)**								
Dunne *et al.*, 2025^22,e^	Sulopenem 1 g IV q24h for 5 days, followed by sulopenem etzadroxil/probenecid 1000 mg PO q12h (Median 9 days)	Ertapenem 1 g IV q24h for 5 days, followed by either Ciprofloxacin 500 mg PO q12h + Metronidazole 500 mg PO q6h or Amoxicillin/Clavulanate 875 mg PO q12h (Median 9 days)	Non-inferiority	Bulgaria, Ukraine, Georgia	micro-MITT: SUL 249; ETP 266	Clinical response (Day 28); 85.5% versus 90.2%, RD: −4.7 (95% CI: −10.3 to 1.0) Non-inferiority not met	*E. coli*, *K. pneumoniae*, *P. aeruginosa*	3GCR-E, CR-E, CR-P
Sun *et al.*, 2022^34,e,f^	Ceftolozane/Tazobactam 1.5 g IV q8h + Metronidazole 0.5 g IV q8h (Mean 5.7 days, SD 2.6)	Meropenem 1 g IV q8h + Placebo (Mean 5.9 days [SD 2.7])	Non-inferiority	China	CE: C-T + MTZ 107; MEM 114	Clinical cure (Day 28); 95.2% versus 93.1%, RD: 2.1 (95% CI −4.7 to 8.8) Non-inferiority met	*E. coli*, *K. pneumoniae*, *P. aeruginosa*	3GCR-E, CR-E, CR-P
IGNITE4: Solomkin *et al.*, 2019^35,e,f^	Eravacycline 1 mg/kg IV q12h + Placebo	Meropenem 1 g IV q8h + Placebo	Non-inferiority	Bulgaria, Ukraine, Latvia	micro-ITT: ERV 195; MEM 205	Clinical cure (Day 25–31); 90.8% versus 91.2%, RD: −0.5 (95% CI −6.3 to 5.3) Non-inferiority met	*E. coli*, *K. pneumoniae*, *P. aeruginosa*	3GCR-E, CR-E
Qin *et al.*, 2017^36,e,f^	Ceftazidime/Avibactam 2.5 g IV q8h + Metronidazole 500 mg IV q8h (Mean 6.9 days, SD 2.9)	Meropenem 1 g IV q8h + Placebo (Mean 7.3 days, SD 2.8)	Non-inferiority	China, Korea, Vietnam	CE: CZA + MTZ 177; MEM 184	Clinical cure (Day 28–35); 93.8% versus 94.0%, RD: −0.2 (95% CI −5.53 to 4.97), *P* < 0.01 Non-inferiority met	*E. coli*, *K. pneumoniae*, *P. aeruginosa*	3GCR-E, CR-E, CR-P
IGNITE-1: Solomkin *et al.*, 2017^37,e,f^	Eravacycline 1 mg/kg IV q12h (Mean 7.6 days, SD 2.8)	Ertapenem 1 g IV q24h + Placebo (Mean 7.6 days, SD 2.4)	Non-inferiority	USA, Argentina, Russia	modified-ITT: ERV 270; ETP 268 micro-ITT: ERV 220; ETP 226	Clinical response (Day 25–31); modified-ITT: 87.0% versus 88.8%, RD: −1.8 (95% CI −7.4 to 3.8) micro-ITT: 86.8% versus 87.6%, RD: −0.8 (95% CI −7.1 to 5.5) Non-inferiority met for all the primary analyses	*E. coli*, *K. pneumoniae*, *P. aeruginosa*	3GCR-E, CR-E, CRAB
RECLAIM 1–2: Mazuski *et al.*, 2016^38,e,f^	Ceftazidime/Avibactam 2.5 g IV q8h Metronidazole 500 mg IV q8h (Mean 8.0 days, SD 3.3)	Meropenem 1 g IV q8h + Placebo (Mean 8.3 days, SD 3.1)	Non-inferiority	NA^b^	micro-MITT: CZA + MTZ 413; MEM 410	Clinical cure (Day 28–35); 81.6% versus 85.1%, RD: −3.5 (95% CI −8.6 to 1.6) Non-inferiority met	*E. coli*, *K. pneumoniae*, *P. aeruginosa*	3GCR-E, CR-E
ASPECT-cIAI: Solomkin *et al.*, 2015^39,e,f^	Ceftolozane/Tazobactam 1.5 g IV q8h + Metronidazole 500 mg IV q8h	Meropenem 1 g IV q8h + Placebo	Non-inferiority	NA^b^	micro-ITT: C-T 389; MEM 417	Clinical cure (Day 24–32); 83.0% versus 87.3%, RD: −4.2 (95% CI −8.9 to 0.5) Non-inferiority met	*E. coli*, *K. pneumoniae*, *P. aeruginosa*	3GCR-E
Chen *et al.*, 2013^40,d^	Moxifloxacin 400 mg IV q24h, followed by 400 mg PO q24h	Ampicillin/Sulbactam 1.5 g IV q6h, followed by 750 mg PO q12h	Non-inferiority	Taiwan	CE: MXF 61; SAM 55	Clinical response (Day 10–14 after EOT); 88.5% versus 83.6%, RD: 5 (95% CI −8% to 18%) Non-inferiority met	*E. coli*, *K. pneumoniae*, *P. aeruginosa*	3GCR-E
Lucasti *et al.*, 2013^41,e,f^	Ceftazidime/Avibactam 2.5 g IV q8h + Metronidazole 500 mg IV q8h (Median 6.0 days)	Meropenem 1 g IV q8h + Placebo (Median 6.5 days)	NA	NA^b^	ME: CZA + MTZ 68; MEM 76	Clinical response (Day 14 after EOT); 91.2% versus 93.4%, RD: −2.2 (95% CI −20.4 to 12.2)	*E. coli*, *K. pneumoniae*, *P. aeruginosa*	3GCR-E^c^, CR-E^g^, CR-P^g^
PROMISE: De Waele *et al.*, 2013^42,e,f^	Moxifloxacin 400 mg IV q24h + Placebo (Mean 7.0 days, SD 2.5)	Ertapenem 1 g IV q24h + Placebo (Mean 6.8 days, SD 2.2)	Non-inferiority	NA^b^	PP: MXF 352; ETP 347	Clinical success (Day 21–28 after EOT); 89.5% versus 93.4%, RD: −3.9 (95% CI −7.9 to 0.4) Non-inferiority met	*E. coli*	3GCR-E
Chen *et al.*, 2010^43^	Tigecycline 50 mg IV q12h (Median 6 days)	Imipenem/Cilastatin 1 g IV q8h (Median 6 days)	NA	China	m-MITT: TGC 60; IPM 55 ME: TGC 52; IPM 48	Clinical response (Day 12–37 days after EOT); m-MITT: 81.7% versus 90.9%, RD: −9.2 (95% CI −23.4 to 4.9) ME: 86.5% versus 97.9%, RD: −11.4 (95% CI −23.5 to 0.7)	*E. coli*	c
Towfigh *et al.*, 2010^44^	Tigecycline 50 mg IV q12h	Ceftriaxone 2 g IV q24h + Metronidazole 1–2 g/day IV	Non-inferiority	USA, Canada, Brazil	CE: TGC 189; CRO + MTZ 187	Clinical cure (Day 10–21 after EOT); 70.4% versus 74.3%, RD: −4.0 (95% CI −13.1 to 5.1), *P* < 0.01 Non-inferiority met	*E. coli*, *K. pneumoniae*, *P. aeruginosa*	
Qvist *et al.*, 2009^45^	Tigecycline 50 mg IV q12h (Mean 6.97 days, SD 3.0)	Ceftriaxone 2 g IV q24h + Metronidazole 500 mg IV q8h (Mean 6.93 days, SD 2.7)	Non-inferiority	NA^b^	CE: TGC 198; CRO + MTZ 189	Clinical cure (Day 8–44 after EOT); 81.8% versus 79.4%, RD: 1.6 (95% CI −6.4 to 9.6) Non-inferiority met	*E. coli*, *K. pneumoniae*, *P. aeruginosa*	
Lucasti *et al.*, 2008^46,e,f^	Doripenem 500 mg IV q8h + Placebo (Mean 6.8 days, SD 3.1)	Meropenem 1 g IV q8h + Placebo (Mean 6.6 days, SD 2.9)	Non-inferiority	NA^b^	micro-MITT: DOR 226; MEM 228 ME: DOR 163; MEM 156	Clinical cure (Day 21–60 after EOT); micro-MITT: 77.9% versus 78.9%, RD: 1.0 (95% CI −9.7 to 7.7) ME: 85.9% versus 85.3%, RD: 0.6 (95% CI −7.7 to 9.0) Non-inferiority met for all the primary analyses	*E. coli*, *P. aeruginosa*, *K. pneumoniae*	
Garbino *et al.*, 2007^47^	Cefepime 2 g IV q12h + Metronidazole 500 mg IV q8h (Mean 8 days, SD 2.8)	Imipenem/Cilastatin 500 mg IV q6h (Mean 9 days, SD 2.7)	Non-inferiority	Switzerland	ITT: FEP + MTZ 60; IPM 61	Clinical cure (Day 5); 87% versus 73%, RD: 15 (95% CI 7 to 29), *P* < 0.01 Non-inferiority met	*E. coli*, *P. aeruginosa*, *P. mirabilis*	NA
Namias *et al.*, 2007^48,e,f^	Ertapenem 1 g IV q24h + Placebo (Mean 7.0 days, SD 3.6)	Piperacillin/Tazobactam 3.375 g IV q6h (Mean 7.6 days, SD 4.0)	Non-inferiority	USA	ME: ETP 123; TZP 108	Clinical response (Day 14 after EOT); 82.1% versus 81.7%, RD: 0.3 (95% CI −9.6 to 10.5) Non-inferiority met	*E. coli*, *K. pneumoniae*, *P. aeruginosa*	
Malangoni *et al.*, 2006^49,e,f^	Moxifloxacin 400 mg IV q24h + Placebo	Piperacillin/Tazobactam 3.375 g IV q6h	Non-inferiority	USA, Canada, Israel	Efficacy-valid population: MXF 183; TZP + AMC 196	Clinical response (Day 25–50); 80% versus 78%, RD: 2 (95% CI −7.4 to 9.3) Non-inferiority met	*E. coli*, *P. aeruginosa*, *K. pneumoniae*	NA
OASIS I: Dela Pena *et al.*, 2006^50^	Ertapenem 1 g IV q24h (Median 6 days)	Piperacillin/Tazobactam 3.375 g IV q6h (Median 6 days)	Non-inferiority	NA^b^	CE-ME: ETP 119; TZP 114	Clinical response (Day 14 after EOT); 89.9% versus 93.9%, RD: −3.9 (95% CI −11.5 to 3.4) Non-inferiority met	*E. coli*, *P. aeruginosa*, *K. pneumoniae*	NA^c^
Oliva *et al.*, 2005^51,e,f^	Tigecycline 50 mg IV q12h + Placebo (Mean 8.1 days, SD 2.8)	Imipenem/Cilastatin 500 mg IV q6h (Mean 7.9 days, SD 2.7)	Non-inferiority	NA^b^	Micro-MITT: TGC 309; IPM 312 ME: TGC 247; IPM 255	Clinical cure (Day 14–35 after EOT); 73.5% versus 78.2%, RD: −4.7 (95% CI −11.8 to 2.6), *P* < 0.01 ME: 80.6% versus 82.4%, RD: −1.8 (95% CI −9.0 to 5.4), *P* < 0.01 Non-inferiority met for all the primary analyses	*E. coli*, *K. pneumoniae*, *P. aeruginosa*	c
Erasmo *et al.*, 2004^52^	Piperacillin/Tazobactam 4.5 g IV q8h (Mean 5.6 days, SD 2.0)	Imipenem/Cilastatin 1 g IV q6h (Mean 5.5 days, SD 2.1)	Non-inferiority	Philippines, China, Korea	CE: TZP 111; IPM 103	Clinical success (Day 28); 97.3% versus 97.1%, *P* = 1.00 Non-inferiority met	*E. coli*, *K. pneumoniae*, *Enterobacter spp*	NA^c^
Solomkin *et al.*, 2003^53,e,f^	Ertapenem 1 g IV q24h + Placebo (Mean 7.6 days, SD 3.0)	PIperacillin/Tazobactam 3.375 g IV q6h (Mean 7.8 days, SD 2.8)	Non-inferiority	NA^b^	ME: ETP 203; TZP 193	Clinical cure (Day 28–42 after EOT); 86.7% versus 81.2%, RD: 5.5 (95% CI −2.2 to 13.1) Non-inferiority met	*E. coli*, *K. pneumoniae*, *P. aeruginosa*	c
Solomkin *et al.*, 2001^54,e,f^	Clinafloxacin 200 mg IV q12h + Placebo (Range 4–14 days)	Imipenem/Cilastatin 500 mg IV q6h (Range 4–14 days)	NA	USA, Canada	Valid Patients: CLX 150; IPM 162	Clinical success; 82% versus 80%, (95% CI −7.0 to 10.0)	*E. coli*, *K. pneumoniae*, *P. aeruginosa*	NA
Torres *et al.*, 2000^55^	Cefminox 2 g IV q12h (Mean 6.3 days, SD 2.8)	Gentamicin 80 mg IV q8h + Metronidazole 500 mg IV q8h (Mean 6.3 days, SD 2.8)	NA	Spain	CE: CMX 76; MET + GEN 76	Clinical cure; 98.6% versus 92.1%, RR: 1.07 (95% CI 1.00 to1.15), *P* > 0.05	*E. coli*, *K. pneumoniae*, *P. mirabilis*	NA
**Complicated urinary tract infection (cUTI)**								
CERTAIN-1: Wagenlehnerl *et al.*, 2024^56,e,f^	Cefepime/Taniborbactam 2.5 g IV q8h + Placebo (Median 7 days, Range 1–15)	Meropenem 1 g IV q8h + Placebo (Median 7 days, Range 2–15)	Superiority, Non-inferiority	Bulgaria, Ukraine, Russian Federation	micro-ITT: FEP-TAN 293; MEM 143	Clinical and microbiological success (Day 19–23); 70.6% versus 58.0%, RD: 12.6 (95% CI 3.1 to 22.2), *P* < 0.01 Non-inferiority met Superiority met	*E. coli*, *K. pneumoniae*, *P. mirabilis*	3GCR-E^c^
Dunne *et al.*, 2023^57,e,f^	Sulopenem 1000 mg IV q24h followed by Sulopenem etzadroxil/probenecid 1000 mg PO q12h ± Placebo (Median 5 days for IV)	Ertapenem 1 g IV q24h followed by Ciprofloxacin 500 mg or Amoxicillin/Clavulanate 875 mg PO q12h ± Placebo (Median 6 days for IV)	Non-inferiority	Russia, Bulgaria, Ukraine	micro-MITT: SUL 444; ETP 440	Composite of clinical cure and microbiological eradication (Day 21); 67.8% versus 73.9%, RD: −6.1 (95% CI −12.0 to −0.1) Non-inferiority not met	*E. coli*, *K. pneumoniae*, *P. mirabilis*	3GCR-E
ALLIUM: Kaye *et al.*, 2022^58,e^	Cefepime/Enmetazobactam 2.5 g IV q8h (Median 8.0 days, Range 7–14)	Piperacillin/tazobactam 4.5 g IV q8h (Median 8.0 days, Range 7–14)	Non-inferiority	Ukraine, Russia, Bulgaria	primary analysis set: FEP-ENM 345; TZP 333	Composite of clinical cure and microbiological eradication (Day 14); 79.1% versus 58.9%, RD: 21.2 (95% CI 14.3 to 27.9), *P* < 0.01 Non-inferiority met	*E. coli*, *K. pneumoniae*, *P. mirabilis*	c
ADAPT-PO: Eckburg *et al.*, 2022^59,e,f^	Tebipenem 600 mg IV q8h + Placebo (Mean 8.7 days, SD 1.8)	Ertapenem 1 g IV q24h + Placebo (Mean 8.5 days, SD 1.9)	Non-inferiority	Ukraine, Russia, Georgia	micro-ITT: TBP 449; ETP 419	Composite of clinical cure and microbiological response (Day 19); 58.8% versus 61.6%, RD: −3.3 (95% CI −9.7 to 3.2) Non-inferiority met	*E. coli*, *K. pneumoniae*, *P. mirabilis*	3GCR-E^c^
FOREST: Sojo-Dorado *et al.*, 2022^60,d^	Fosfomycin 4 g IV q6h (Mean 5.4 days, SD 0.9)	Ceftriaxone 1 g IV q24h or Meropenem 1 g IV q8h (Mean 5.5 days, SD 1.8)	Non-inferiority	Spain	modified-ITT: FOS 70; CRO or MEM 73	Clinical and microbiological cure (Day 5–7 after EOT); 68.6% versus 78.1%, RD: −9.4 (95% CI −21.5 to ∞), *P* = 0.10 Non-inferiority not met	*E. coli*	3GCR-E (*E. coli*)^c^
ZEUS: Kaye *et al.*, 2019^61,e^	Fosfomycin 6 g IV q8h (Median 7.1 days)	Piperacillin/Tazobactam 4.5 g IV q8h (Median 7.1 days)	Non-inferiority	NA^b^	micro-MITT: FOS 184; TZP 178	Composite of clinical cure and microbiological eradication (Day 19–21); 64.7% versus 54.5%, RD: 10.2 (95% CI −0.4 to 20.8) Non-inferiority met	*E. coli*, *K. pneumoniae*, *P. mirabilis*	3GCR-E, CR-E^c^
EPIC: Wagenlehner *et al.*, 2019^62,e,h^	Plazomicin 15 mg/kg IV q24h (Mean 9.2 days)	Meropenem 1 g IV q8h (Mean 8.9 days)	Non-inferiority	NA^b^	micro-MITT: PLZ 191; MEM 197	Composite of clinical cure and microbiological eradication (Day 5, Day 15–19 after EOT); Day 5: 88.0% versus 91.4%, RD: −3.4 (95% CI −10.0 to 3.1) Day 15–19 after EOT: 81.7% versus 70.1%, RD: 11.6 (95% CI 2.7 to 20.3) Non-inferiority met for all the primary analyses	*E. coli*, *K. pneumoniae*, *E. cloacae*	3GCR-E, CR-E^c^
APEKS-cUTI: Portsmouth *et al.*, 2018^63,e^	Cefiderocol 2 g IV q8h (Mean 9.0 days, SD 2.7)	Imipenem/Cilastatin 1 g IV q8h (Mean 9.0 days, SD 2.6)	Non-inferiority	Russia, Europe, Japan	modified-ITT: FDC 252; IMP 119	Composite of clinical and microbiological response (Day 5–9 after EOT); 73% versus 55%, RD: 18.6 (95% CI 8.2 to 28.9), *P* < 0.01 Non-inferiority met	*E. coli*, *K. pneumoniae*, *P. aeruginosa*	3GCR-E^c^
TANGO I: Kaye *et al.*, 2018^64,e,f^	Meropenem/Vaborbactam 4 g IV q8h + Placebo (Median 10.1 days, range 1–17)	Piperacillin/Tazobactam 4.5 g IV q8h + Placebo (Median 9.9 day, range 2–15)	Non-inferiority	Ukraine, Belarus, Bulgaria	micro-MITT: MEV 192; TZP 182	FDA primary endpoint: Overall success (EOT); 98.4% versus 94.0%, RD: 4.5 (95% CI 0.7 to 9.1), *P* < 0.01 EMA primary endpoint: Microbial eradication (Day 7 after EOT); 66.7% versus 57.7%, RD: 9.0 (95% CI −0.9 to 18.7), *P* < 0.01 Non-inferiority met for all the primary analyses	*E. coli*, *K. pneumoniae*, *P. mirabilis*	3GCR-E, CR-E^g^
RECAPTURE: Wagenlehner *et al.*, 2016^65,e,f^	Ceftazidime/Avibactam 2.5 g IV q8h (Median 7 days)	Doripenem 500 mg IV q8h + Placebo (Median 8 days)	Non-inferiority	Ukraine, Russia, Romania	micro-MITT: CZA 393; DOR 417	FDA Co-primary Endpoints: Symptomatic resolution (Day 5), Combined symptomatic resolution and microbiological eradication (Day 21–25); Endpoint 1: 70.2% versus 66.2%, RD: 4.0 (95% CI −2.4 to 10.4) Endpoint 2: 71.2% versus 64.5%, RD: 6.7 (95% CI 0.3 to 13.1) EMA Primary Endpoint: Microbiological eradication (Day 21–25); 77.4% versus 71.0%, RD: 6.4 (95% CI 0.3 to 12.4) Non-inferiority met for all the primary analyses	*E. coli*, *K. pneumoniae*, *P. aeruginosa*	3GCR-E^c^
ASPECT-cUTI: Wagenlehner *et al.*, 2015^66,e,f^	Ceftolozane/Tazobactam 1.5 g IV q8h	Levofloxacin 750 mg IV q24h + Placebo	Non-inferiority	Russia, Ukraine, Poland	micro-MITT: C-T 398; LEV 402	Composite of clinical cure and microbiological eradication (Day 5–9 after EOT); 76.9% versus 68.4%, (95% CI 2.3 to 14.6) Non-inferiority met	*E. coli*, *K. pneumoniae*, *P. mirabilis*	3GCR-E, QR-GN
Park *et al.*, 2012^67,e^	Ertapenem 1 g IV q24h	Ceftriaxone 2 g IV q24h	Non-inferiority	South Korea	ME: ETM 66; CRO 71	Microbiological response (Day 5–9 after EOT); 87.9% versus 88.7%, RD: −0.8 (95% CI −11.7 to 10.2)	*E. coli*, *K. pneumoniae*, *C. freundii*	3GCR-E^c^
Vazquez *et al.*, 2012^10^	Ceftazidime/Avibactam 625 mg IV q8h + Metronidazole 500 mg IV q8h (Median 11 days)	Imipenem/Cilastatin 500 mg IV q6h (Median 12 days)	NA	USA	ME: CZA 27; IPM 35	Microbiological response (Day 5–9 after EOT); 70.4% versus 71.4%, RD: 1.1 (95% CI −27.2 to 25.0)	*E. coli*, *P. aeruginosa*, *P. mirabilis*	3GCR-E^c^
DORI-05–06: Redman *et al.*, 2010^68,e^	Doripenem 500 mg IV q8h	Levofloxacin 250 mg IV q24h	Non-inferiority	NA^b^	micro-MITT: DOR 664; LVX 321 ME: DOR 530; MER 265	Microbiological response (Day 5–11 after EOT); micro-MITT:80.9% versus 78.2%, RD: 2.7 (95% CI −3.0 to 8.3) ME: 82.8% versus 83.4%, RD: −0.6 (95% CI −6.4 to 5.2) Non-inferiority met for all the primary analysis	*E. coli*, *K. pneumoniae*, *P. mirabilis*	QR-GN
Naber *et al.*, 2009^69,e,f^	Doripenem 500 mg IV q8h + Placebo (Mean 9.5 days)	Levofloxacin 250 mg IV q24h + Placebo (Mean 9.1 days)	Non-inferiority	NA^b^	ME: DOR 280; LVX 265	Microbiological cure (Day 5–11 after EOT); 82.1% versus 83.4%, RD: −1.3 (95% CI −8.0 to 5.5) Non-inferiority met	*E. coli*, *P. mirabilis*, *K. pneumoniae*	QR (levofloxacin)
Klausner *et al.*, 2007^70,e,f^	Levofloxacin 750 mg IV q24h + Placebo	Ciprofloxacin 400 mg IV or 500 mg PO q12h	Non-inferiority	USA	MITT: LVX 94; CIP 98 ME: LVX 80; CIP 76	Microbiological eradication (Day 15–19); MITT: 83.0% versus 79.6%, RD: −3.4 (95% CI −14.4 to 7.6) ME: 92.5% versus 93.4%, RD: 0.9 (95% CI −7.1 to 8.9) Non-inferiority met	*E. coli*, *K. pneumoniae*, *K. oxytoca*	QR^g^
Carmignani *et al.*, 2005^71,e,f^	Prulifloxacin 600 mg PO q24h + Placebo	Ciprofloxacin 500 mg PO q12h	Non-inferiority	Italy, France	ITT: PRU 98; CIP 108	Microbiological response (Day 5–7); 90.8% versus 77.8%, (lower limit one-tailed 95% CI 4.9) Non-inferiority met	*E. coli*, *P. mirabilis*, *K. pneumoniae*	QR^g^
Tomera *et al.*, 2002^72,e,f^	Ertapenem 1 g IV q24h + Placebo (Mean 11.7 days, SD 2.2)	Ceftriaxone 1 g IV q24h + Placebo (Mean 11.8 days, SD 2.2)	Equivalence	NA^b^	ME: ETM 159; CRO 171	Microbiological eradication (Day 5–9); 91.8% versus 93.0%, RD: −1.2 (95% CI −7.6 to 5.1) Equivalence met	*E. coli*, *K. pneumoniae*, *P. mirabilis*	c
Jimenez-Cruz *et al.*, 2002^73,e^	Ertapenem 1 g IV q24h (Mean 12.6 days, SD 2.0)	Ceftriaxone 1 g IV q24h (Mean 12.7 days, SD 1.8)	Equivalence	NA^b^	Microbiologically assessable population: ETM 97; CRO 53	Microbiological eradication (Day 5–9 after EOT); 85.6% versus 84.9%, RD: 0.6 (95% CI −12.9 to 14.1) Equivalence met	*E. coli*, *K. pneumoniae*	c
Cox *et al.*, 2002^74,e,f^	Gatifloxacin 400 mg PO q24h + Placebo	Ciprofloxacin 500 mg PO q12h	NA	USA	ME: GAT 91; CIP 95	Bacteriological response rate (Day 4–9 after EOT); 92% versus 83%, RD: 9.0 (95% CI −4.1 to 24.5)	*E. coli*, *K. pneumoniae*, *P. aeruginosa*	

ATM-AVI, aztreonam/avibactam; BAT, best available therapy; CE, clinically evaluable; CE-ME, clinically and microbiologically evaluable; CFM, cefminox; CFO/SUL, cefoperazone/sulbactam; CIP, ciprofloxacin; CLX, clinafloxacin; CR-E, CR-P, carbapenem-resistant *P. aeruginosa*; CRO, ceftriaxone; CST, Colistin; C-T, ceftolozane/tazobactam; CZA, ceftazidime/avibactam; DOR, doripenem; EE, efficacy evaluable; EOT, end of treatment; ERV, eravacycline; ETM, ertapenem; FDC, cefiderocol; FEP, cefepime; FEP-ENM, cefepime/enmetazobactam; FEP-TAN, cefepime/taniborbactam; FOS, fosfomycin; GAT, gatifloxacin; IPM, imipenem/cilastatin; I-R, imipenem/relebactam; ITT, intention-to-treat; LVX, levofloxacin; MBL-GN, metallo-β-lactamase producing Gram-negative; ME, microbiologically evaluable; MEM, meropenem; MEV, meropenem/vaborbactam; MTZ, metronidazole; micro-ITT, microbiological intent-to-treat; micro-MITT, microbiologically modified intention-to-treat population; MITT, modified intention-to-treat population; MXF, moxafloxacin; PLZ, plazomicin; PP, per protocol; PRU, prulifloxacin; QR-GN, quinolone resistant Gram-negatives; RD, risk difference; RIF, rifampicin; RR, relative risk; SUL, sulopenem; SUL/DUR, sulbactam/durlobactam; TBP, tebipenem; TGC, tigecycline; TOC, test of cure; TZP, piperacillin/tazobactam.

^a^Studies that did not declared a primary hypothesis are listed as NA.

^b^More countries were listed but no enrolment numbers were provided.

^c^CR pathogen listed among exclusion criteria.

^d^Non-industry funded trial (i.e. federal or other sources of grant funding).

^e^Double-blind design.

^f^Use of placebo.

^g^Fewer than five isolates reported.

^h^Both industry and non-industry funding.

^i^Defined by the authors as those resistant to two or more classes of antibiotics and high-risk organisms (defined as *Pseudomonas* spp., methicillin-resistant *S. aureus*, *S. maltophilia*, *Acinetobacter* spp. and MDR bacteria).

### Baseline characteristics of included trials

**Figure 2.**
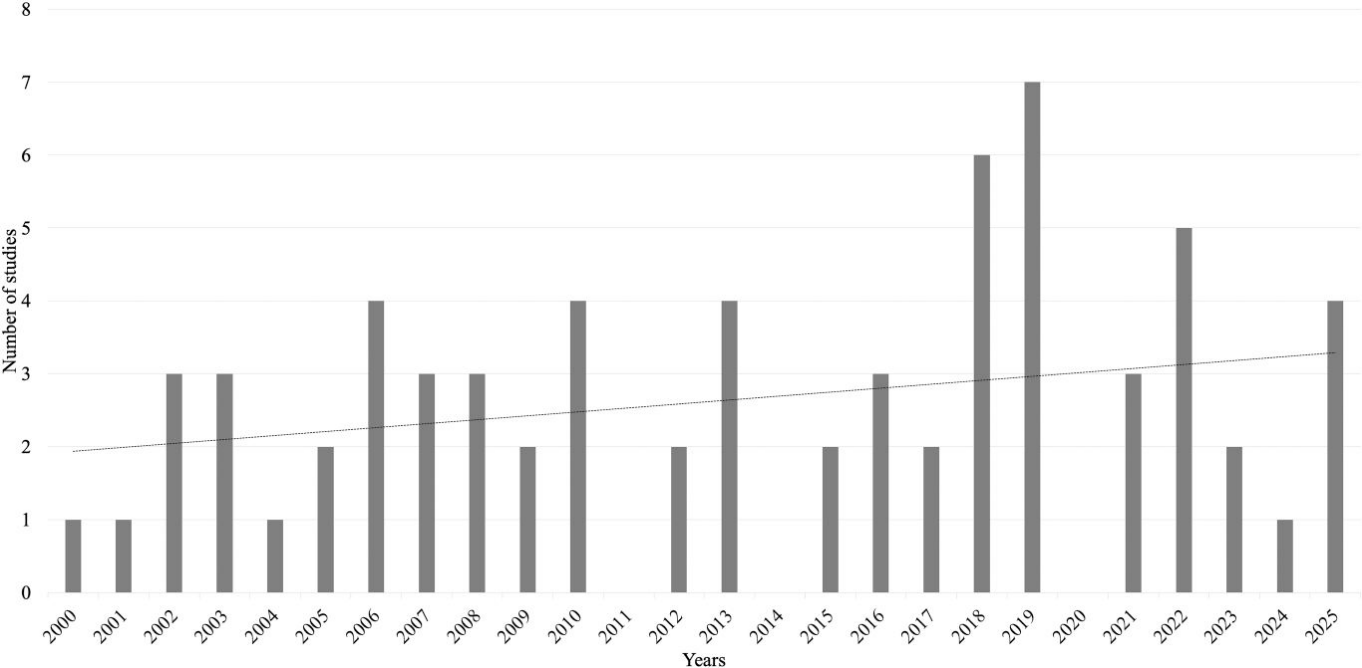
Number of included studies per year, 2000–2025. The number of RCTs published each year are represented by the grey bars. The black dotted line represents publication trend over time.

Publications were evenly distributed across the study period (*P* = 0.250). A total of 27 (39.7%) articles were published between 2000 and 2010, 26 (38.2%) between 2011 and 2020, and 14 (22.4%) between 2021 and 2025. The year with the highest number of published RCTs was 2019 (7 studies) (Figure 2).

Figure S1 (available as Supplementary data at *JAC-AMR* Online) shows the distribution of top-enrolling countries (sufficient data provided in 43 studies). Ukraine was the most frequent ‘top three’ enrolling country (14/43 RCTs, 32.6%), followed by Russia (10, 23.3%) and the USA (10, 23.3%). Fifty-nine studies (86.8%) were industry funded. Seven studies reported non-industry funding sources (e.g. federal grants) and in one study no funding source was reported (Table 1).

**Figure 3.**
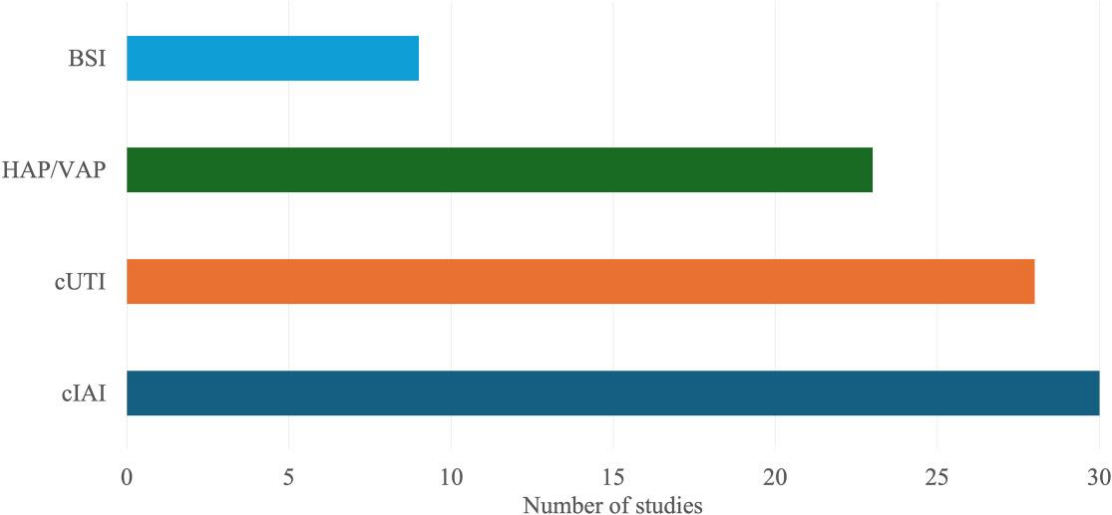
Bar graph showing number of published studies according to each clinical syndrome. Some studies included more than one syndrome.

Most studies targeted a single clinical syndrome, with 11 RCTs (16.2%) targeting more than one. Overall, cIAIs and cUTIs were the most frequent infections studied [in 30 (44.1%) and 28 (41.2%) RCTs, respectively], followed by HAP/VAP and BSI (Figure 3). Twelve RCTs targeted one or more specific pathogen, seven of which supported regulatory submissions. Nine of the 12 studies (75.0%) targeted more than one pathogen, whereas three (25.0%) addressed a single pathogen, *Acinetobacter baumannii* in two studies and *Escherichia coli* in one.

### Study design

Forty-two (61.8%) studies employed a double-blind design, and among these 30 (44.1%) involved placebo administration (Table 1). Two different placebo strategies were generally adopted. In a single study, placebo administration was provided only in the non-interventional study arm (with the same background therapy administered to both study arms),^16^ and in the 29 other studies, a dummy-placebo was administered to both study groups (e.g. due to differences in infusion time or administration schedule between the study and control drug).

Fifty-six studies (82.4%) declared a pre-specified hypothesis to be tested. Among these, non-inferiority designs were the most common (48/56 RCTs, 85.7%). The margin for the 95% confidence interval (CI) for the outcome difference applied to declare non-inferiority varied across the studies ranging from 5% adopted in the MERINO trial,^20^ to 20% sed in six other trials,^15,28,33,52,63,67^ with a mode of 10% in 17 studies (out of total of 48 non-inferiority trials, 35.4%).^23–25,37–40,42,47,49,57,58,65,66,68,69,75^ Superiority of investigational over control treatment was assessed as the primary outcome in only four studies (5.9%).^16,17,19,29^ The most frequent primary outcome used was clinical (e.g. clinical success) in 39 (57.4%) studies followed by a combination of clinical and microbiological outcomes (13 studies, 19.1%), all-cause mortality (12, 17.6%) and a microbiological outcome (12, 17.6%), (Table 1). All-cause mortality (12 studies) was primarily assessed in studies enrolling patients with BSI or HAP/VAP. Indeed, only three studies that included patients with cIAI and cUTI adopted this primary outcome. In contrast, all studies that exclusively enrolled patients with cIAI used clinical success or failure as the primary outcome (23 studies), whereas purely microbiological outcomes were considered only in cUTI studies (11 studies) and in RCTs involving multiple clinical indications, including cUTI (1 study).

A large degree of heterogeneity was observed regarding the time points at which primary outcomes were assessed. The earliest evaluation was adopted by Wagenlehner *et al.* in two studies of cUTI (day 5 of therapy).^62,65^ The latest primary outcome evaluation was used by Lucasti *et al.* in a study of cIAI (60 days after the completion of therapy).^46^ When the primary outcome was all-cause mortality, time points were more consistent, with 11 of 12 assessing mortality on day 28–30 and one study assessing it on day 14.^22^

### Study population

The median size of the primary outcome study population was 361.5 (IQR 190.75–512) participants. The largest study enrolled 985 participants, whereas the smallest enrolled 15 (included due to its use for regulatory submission). Most studies had exclusion criteria involving immunocompromising conditions (40, 58.8%). Patients with sepsis and/or septic shock were excluded in 19 studies (27.9%) (Table 1). APACHE II score was listed among exclusion criteria in 27 studies (39.7%) with an upper limit set to 30 in 22/26 studies (84.6%). An exclusion criterion based on creatinine clearance was adopted in 33 (48.5%) studies. The most frequent lower limit of acceptable creatinine clearance was 30 mL/min in 12/33 studies (36.4%), followed by 15 mL/min and 20 mL/min in 6 (18.2%) and 4 (12.1%) studies, respectively. A history of epilepsy or a condition predisposing to epilepsy or seizures was listed as exclusion criteria in 16 studies (23.5%).

### Characteristics of investigational and control drugs

A total of 29 unique antibiotics were evaluated. Novel agents—defined as drugs not previously approved or available on the market for fewer than 5 years at the time of RCT publication—were evaluated in most studies (43, 63.2%) (Table 1). β-lactams were the most frequent class of antibiotics studied (46 RCTs, 67.6%), followed by fluoroquinolones (9, 13.2%) and tetracyclines (7, 10.3%). At the individual drug level, ceftazidime/avibactam was the most frequent investigational agent studied (7 RCTs, 10.3%), followed by ertapenem (6 RCTs, 8.8%) and tigecycline (5 RCTs, 7.4%).

Fifteen unique antibiotics were used in control arms. β-lactams were most commonly used (41, 60.3%), the most frequent agent being meropenem (16 RCTs, 23.5%) followed by imipenem/cilastatin (12 RCTs, 17.6%) and piperacillin/tazobactam (9 RCTs, 13.2%). Best available therapy (BAT) was used as control therapy in four studies (5.9%).

### Microbiology and antimicrobial resistant pathogens

In 46 RCTs (67.6%, including 23 cIAI, 20 cUTI, one BSI and two involving multiple clinical syndromes), *E. coli* was the most frequently isolated pathogen, followed by *K. pneumoniae* (eight RCTs, 11.8%, including five HAP/VAP and three involving multiple clinical syndromes) and *Pseudomonas aeruginosa* (five RCTs, 7.4%, including four HAP/VAP and one involving multiple clinical syndromes). When evaluating the top three most frequently isolated pathogens in a study, *K. pneumoniae* was most often in the top three (57 RCTs, 83.8%), followed by *E. coli* (51 RCTs, 75.0%) and *P. aeruginosa* (25 RCTs, 36.8%) (Table 1).

With regards to MDR pathogens, 28 studies reported data including third-generation cephalosporin-resistant *Enterobacterales*. Carbapenem-resistant pathogens were reported in 26 RCTs (38.2%) and CRAB specifically in nine RCTs (13.2%). A description of mechanisms mediating antimicrobial resistance was reported in 30 studies (44.1%). The most commonly reported mechanism was extended-spectrum β-lactamase (ESBL) production (25/30, 83.3%). Carbapenemase production was reported in 12 studies (40.0%) [serine-carbapenemase-producers (i.e. KPCs) were reported in nine (30.0%) and metallo-beta-lactamases (MBLs) in nine RCTs (30.0%)].

Twelve of 68 (17.6%) studies focused specifically on MDR pathogens: 3/12 (25.0%) on third-generation cephalosporin-resistant pathogens and/or ESBL-producers^18,20,60^; 9 (75.0%) on carbapenem-resistant pathogens^8,9,11–13,15–17,19^ [of which two (3.1%) focused specifically on carbapenem-resistant *A. baumannii*].^15,19^ Among the seven RCTs primarily targeting inclusion of carbapenem-resistant pathogens, three trials evaluated the superiority of colistin combined with meropenem (AIDA and OVERCOME trials)^16,17^ or rifampicin (Durante-Mangoni *et al.*)^19^ compared to colistin monotherapy. These trials all failed to demonstrate superiority of combination therapy. In the CREDIBLE-CR trial,^9^ cefiderocol was compared with BAT for carbapenem-resistant pathogens and demonstrated similar efficacy in HAP/VAP and BSI, and higher rates of microbiological success in cUTI, although no null hypotheses were pre-defined. The ATTACK trial compared sulbactam/durlobactam with colistin (both arms also included imipenem-cilastatin) for treatment of CRAB and demonstrated non-inferiority of the investigational regimen with respect to all-cause mortality.^15^ Notably, the 95% CI for the difference in mortality (−30.0 to 3.5) in ATTACK did not support superiority, although the secondary outcome of clinical cure rate at test of cure was significantly higher. The TANGO II trial compared meropenem/vaborbactam with BAT in BSI, cUTI, cIAI and HAP/VAP due to carbapenem-resistant GNB.^8^ In TANGO II the overall success rate was higher among those treated with meropenem/vaborbactam, however, no pre-specified null hypothesis was declared. The RESTORE-IMI 1 trial was a double-blind placebo-controlled trial comparing imipenem/relebactam versus colistin plus imipenem in patients with cUTI, cIAI or HAP/VAP due to imipenem-resistant GNB.^13^ Similar overall response rates were reported although no formal hypothesis test was declared. The CARE trial comparing plazomicin with colistin for the treatment of HAP/VAP and/or BSI (both arms also received meropenem or tigecycline)^11^ and the ASSEMBLE trial comparing aztreonam/avibactam (plus metronidazole in patients in the cIAI group) with BAT for the treatment of HAP/VAP, BSI, cUTI and cIAI, were interrupted prematurely.^12^ Both studies reported favourable outcomes in the groups receiving an investigational antibiotic; however, in each study, no null hypothesis was pre-defined or tested.

## Discussion

To the best of our knowledge, this is the first analysis to evaluate RCTs published in the 21st century that focused on treatment of infections caused by GNB. Encouragingly, there has been an increase in the number of RCTs published during this time period, and most studies were of high quality with regards to design and conduct. Most RCTs were industry funded and supported regulatory submissions. Patients were enrolled primarily in eastern Europe, Russia and North America. The main limitations of these trials were a lack of generalizability of findings to real-world scenarios secondary to exclusion of certain patient populations and pathogens, and the primary endpoints used.

There was a consistent increase, even if not statistically significant, in the number of RCTs published annually during the study period (Figure 2), reflecting increased awareness of the MDR GNB threats, as well as new funding opportunities designed specifically to target therapeutic options for these pathogens.^76^ There were numerous publications after 2014, coinciding with FDA and EMA approvals of ceftolozane-tazobactam and ceftazidime-avibactam, which ushered in a new era of therapy for MDR GNB.

Study quality also improved after 2014, with an increase in the proportion of double-blind and placebo-controlled studies. Since 2015, 65.7% of studies were double-blind and >50% included the use of placebo (compared to 57.6% and 45.5%, respectively, published before 2015). These types of design are essential to reduce the risk of biases related to treatment assignment awareness, including detection and performance bias,^77^ particularly when relatively subjective outcomes such as clinical cure are measured.

### Limitations in GNB RCTs published in the 21st century

#### Exclusion of key MDR pathogens

Even though many of the newer antimicrobials were designed specifically to target carbapenem-resistant pathogens, in 34.4% (11/32) of these trials, infections due to carbapenem-resistant pathogens were listed among exclusion criteria. Most of these trials were supportive of a regulatory submission and the control antibiotic used in these trials was not active against carbapenem-resistant pathogens. However, the exclusion of carbapenem-resistant pathogens, the treatment of which represents one of the greatest unmet therapeutic needs in infectious diseases, creates a discrepancy between the real-world target population and RCT participants, leaving readers with little relevant clinical information other than safety data. A critical unmet need is related to the treatment of CRAB and CR-*P. aeruginosa* (CR-P) infections. Only five RCTs specifically targeting these GNB were identified, with three investigating older drugs (e.g. polymyxins).^9,15–17,19^ Trials targeting serious infections due to CRAB and CR-P are difficult to conduct due to enrolment challenges and a high degree of severity of illness among eligible patients. As a result, these trials, many of which are conducted to support regulatory submission, are particularly expensive, and often open label; indeed, 12/32 (37.5%) RCTs of newer agents designed to target MDR pathogens had an open-label design.

#### Most relevant infectious syndromes were not studied and key populations were excluded

Most studies targeted cIAI or cUTI (30 and 28 RCTs, respectively) and in many cases, the antibiotics studied in these trials are used by clinicians to treat HAP/VAP and/or bloodstream infection, where the severity of illness is often greater than in cUTI or cIAI. Across all syndromes (cUTI, cIAI, HAP/VAP and BSI) key populations are often excluded, such as patients with immunocompromising conditions or receiving immunocompromising medications (in almost 60% of studies), patients with sepsis/septic shock (excluded in almost 30% of studies), patients with higher APACHE II score (excluded in almost 40% of studies) and patients with impaired kidney function (a creatinine clearance limit adopted in almost 50% of the studies). Thus, RCTs for new antimicrobials often leave clinicians without data that are generalizable to the patients that they often need to treat with these new agents: those with a high degree of severity of illness and those with HAP/VAP and/or BSI.

#### Regulatory constraints on study design

Regulatory authorities (e.g. FDA and EMA) have had a strong impact on which clinical syndromes are studied and the types of trial design used (e.g. the primary outcome used). Accordingly, in this study primary outcomes largely reflected regulatory constraints. Two relevant examples are related to outcomes emphasized by FDA for HAP/VAP and cUTI trials. The most common primary outcome used in HAP/VAP trials was 28–30 day all-cause mortality. As patients with HAP/VAP are in general a severely ill population, there are often several competing risks for death, and mortality rates are usually high in both treatment groups, making it almost impossible to demonstrate superiority when comparing a therapeutic intervention to a standard of care treatment.^78^ RCTs of cUTI use a primary outcome that does not reflect the real-world clinical practice. The primary outcomes of cUTI studies included in this review always included a microbiological endpoint as either the primary outcome, or a component of the primary outcome. However, a repeat culture from the urinary tract is not part of the standard patient care. In most instances, clinical response was similar between treatment groups, with differences driven almost exclusively by microbiological failure, even though its clinical relevance remains uncertain.^79,80^ In both of these examples, the primary endpoints used made the RCT data difficult to interpret for clinicians and in some cases, did not adequately demonstrate the therapeutic impact attributable to a new agent.

Another issue is the plethora of non-inferiority studies. Of the 68 studies included in this review, only four were designed to demonstrate superiority. In an ideal scenario, every newly developed drug should be tested for superiority compared to the current standard of care. Unfortunately, in many instances, demonstrating superiority is difficult and in some cases, almost impossible.^81^ First, in most cases, testing a novel antimicrobial against placebo is not ethical given the availability of other active treatments. Thus, the comparison against an active control drug usually makes it extremely difficult to reject a null hypothesis especially for a mortality endpoint (e.g. in HAP/VAP) where the relatively small effect size of the investigational drug would require a very large sample size to be detected.^81^ Second, in a superiority trial, when the benefit of the studied intervention is insufficient to demonstrate superiority, the trial is considered a failure by regulatory bodies—even if the investigational agent performed well, making a superiority trial financially risky to undertake—particularly for industry. This consideration is consistent with the evidence that all the included RCTs that used a superiority design were federally funded as opposed to industry funded. Thus, in addition to having RCT results that are of limited generalizability due to constraints related to study population, pathogens included and primary outcomes used, almost all studies of novel agents have a non-inferiority design. In the best-case scenario, RCT results of novel antimicrobials only demonstrate that the new treatment is ‘as good as’ or ‘no better than’ standard of care therapy, and new agents run the risk of being categorized as a ‘me too’ drug.

#### Additional challenges in conducting RCTs for the treatment of carbapenem-resistant GNB

We are now entering an era where an increasing number of agents are becoming available for treatment of carbapenem-resistant GNB. Ideally, studies would be conducted in settings where multiple new active agents are available, such as in the USA and some European countries, so that newer agents can be compared to one another. Unfortunately, due to relatively low rates of infection due to carbapenem-resistant GNB in these countries (e.g. CRAB and CR-*Enterobacterales* in the USA),^82^ enrolment of patients with these types of pathogen is particularly challenging in these settings. In addition, trials comparing two newer agents to one another, both of which demonstrate improved outcomes compared to older antibiotics (e.g. the polymyxins) would require greater enrolment numbers to demonstrate a benefit of one agent over another, making superiority trials particularly challenging to conduct.

Another complicated issue is the choice of comparator therapeutic agents in studies of newer antimicrobials active against carbapenem-resistant GNB. Treatment strategies vary across countries due in part to differences in the availability of newer agents, differences in local guidelines and distinct resistance mechanism epidemiology.^83^ Accordingly, the identification of an acceptable unique treatment strategy for control groups in multinational trials is challenging. The adoption of a BAT strategy that allows investigators to use different therapeutic combinations in line with their local practice has helped in facilitating both the emulation of real word practice and the inclusion of centres from many different countries. However, the impracticality of a double-blind design and the heterogeneity of treatment in a BAT control group complicates the interpretation of study results, as well as determination of treatment effect of investigational agents.

#### Limited funding sources

Of the RCTs included in this study, only seven were entirely funded by a non-industry source, including federal grants. Costs of research and development of a new antimicrobial drug are estimated to be >1 billion dollars and in most cases,^84^ available grants are not large enough to sustain high-quality RCTs involving treatment of MDR GNB. Thus, almost all RCTs of newer antibiotics in this study were funded by pharmaceutical companies.

### The path forward

Many GNB RCTs suffer from a lack of geographic diversity, having traditionally focused on eastern Europe for enrolment. The two most frequent ‘top-enrolling’ countries in the analysed studies were Ukraine and Russia (Figure S1). Unfortunately, the ongoing international conflict involving these countries along with associated sanctions and regulatory constraints have made enrolment for GNB RCTs particularly challenging. Compounding this situation is that, as consequence of war, antimicrobial resistance often increases, further intensifying the need for new agents.^85,86^ To improve the likelihood of consistent patient enrolment despite unforeseen disturbances, future trials should consider geographically diverse sites including those in low-middle income countries, and explore opportunities in underrepresented regions such as Africa and the Middle East. However, the unpredictability and the quick shift of geopolitical balances increase the challenges in these areas as well. In addition, efforts should be made to develop research infrastructures in some of these underserved regions with a high incidence of MDR GNB and to create programmes to facilitate access to novel agents, including providing countries that participate in trials of new antibiotics with affordable access to these drugs.

One way to improve the efficiency of conducting GNB RCTs is through the use of platform trials, which were increasingly used during the COVID-19 pandemic. Platform trials are a type of adaptive trial that allow for multiple interventions to be studied, sometimes simultaneously, using a common study infrastructure. Platform trials often use a master protocol, shared control groups and allow for additions of new treatment arms and removal of ineffective ones during an ongoing trial. In addition to providing an efficient, cost-friendly research framework, this type of trial can provide relevant clinical evidence that can be readily applied into clinical practice.^87–89^

Studies that use conventional clinical trial endpoints are subject to various limitations including the need for large sample sizes, being subject to various biases and failing to adequately capture nuances in patient outcomes and clinical status, such as adverse events, length of stay, readmission and quality of life. Hierarchical composite endpoints (HCE) such as desirability of outcome ranking offer an alternative type of endpoint that may address some of these limitations. Unlike classical composite outcomes, HCE can use a variety of diverse outcome scenarios and a hierarchical ranking system of outcome desirability on the basis of clinical relevance, physician preference and/or patient preference.^90^ The win ratio is an analytic strategy that uses HCEs, to systematically compare each subject in the intervention group with each subject in the control arm to determine the proportion of ‘wins’ (according to HCE) in the intervention arm. Use of HCEs facilitates adequate powering of clinical trials, and in addition to providing an overall comparative favourability analysis between two agents, it provides data on a variety of outcomes that are clinically relevant. HCEs have been used in other medical specialties,^91–93^ and infectious diseases RCTs have begun to use HCEs and the win ratio.^94–96^ We anticipate increased use of HCEs in GNB RCTs in the future, which will help to determine whether these types of endpoint are advantageous over traditional ones.^97–99^

One of the limiting factors in RCTs, including those addressing specific pathogens (and in particular, those with an MDR phenotype), is the time needed to identify a definitive aetiological diagnosis and subsequent antimicrobial susceptibility test results.^100,101^ Withholding treatment for a definite microbiological diagnosis is unethical, and therefore in RCTs patients typically receive off-study empirical antibiotic therapy and are not enrolled until test results are available at which time they have already received 48–72 hours of therapy. This lead-in treatment period before enrolment reduces the ability to measure the maximal impact of an interventional agent. Attempts to enrol patients pre-emptively, before microbiologic tests return, often lead to inclusion of participants who ultimately need to be withdrawn from study treatment once microbiology tests are finalized. Rapid diagnostic tests have been developed that provide pathogen and resistance determinant information rapidly—in some cases, only hours after a specimen is obtained from the patient—which allows for enrolment of patients early in the treatment period when an intervention is likely to have its greatest impact.^101–103^ The use of rapid diagnostic tests in RCTs targeting specific pathogens and/or resistance phenotypes can be particularly useful to facilitate early, efficient enrolment of appropriate patients. This technology has already been used in some GNB RCTs and probably will be more frequently incorporated in future ones.^104^

Target trial emulation provides efficient methods to create simulated study cohorts using data from observational trials, while also controlling for confounding factors. Target trial emulation can effectively estimate causal effects with a high degree of quality. This methodology is an alternative when it is not feasible or ethical to conduct a standard RCT and can also be used to supplement RCTs and other types of clinical study.^105^

Increased funding from non-industry sources is needed to support international trials that address clinically relevant, real-world scenarios. Trials evaluating off-patent agents or conducting head-to-head comparisons of two effective newer agents might be of important clinical relevance but are unlikely to be funded by industry. Such studies will require significant support from federal and foundation sources to be performed. Efforts to streamline regulatory requirements and hurdles will also foster the development of effective international trials.

### Conclusion

There has been a bolus of high-quality RCTs of GNB infection treatment in the 21st century, several involving novel antibiotics. Unfortunately, many do not provide generalizable, real-world data that can be readily interpreted and applied by clinicians. Few studies have focused on MDR GNB, for which there is the great unmet therapeutic need. Changes to the regulatory requirements with regards to study design and primary outcomes would help to foster the production of more clinically relevant RCT data. Increased funding opportunities outside of industry are also needed to support real-world RCTs that target complex populations and resistant pathogens for which clinicians are challenged, with often limited or sub-optimal therapeutic options.

## Supplementary Material

dlag122_Supplementary_Data

## Transparency declarations

F.P. and H.S. have no conflicts of interest to declare. N.N. has speaker honoraria from Astellas. J.M.P. has received grant support from Merck, Shionogi, Abbvie and Innoviva. He has also served as a consultant for GlaxoSmithKline, Q-linea and CorMedix in the past 2 years. E.D.M. received institutional funding, personal speaker fees or advisory board membership honoraria from Pfizer, Angelini, Advanz pharma, Infectopharm, Shionogi, Menarini, Mundipharma, Tillots pharma and Trx in the last 2 years. K.K. served as a consultant for Merck, GlaxoSmithKline, Omnix, Abbvie, Shionogi, Carb-X, ITERUM, Spero and Meitheal.

## Supplementary data

Figure S1 is available as Supplementary data at *JAC-AMR* Online.
