# Supplementary material for: Gram-negative bacterial infection randomized controlled trials (RCTs) in the 21st century: characteristics and evolution
Source: JAC Antimicrob Resist. 2026 Jul 23;8(4):dlag122. doi: 10.1093/jacamr/dlag122 (PMC13395242; doi:10.1093/jacamr/dlag122)
Supplement: dlag122_Supplementary_Data [file dlag122_supplementary_data.docx]

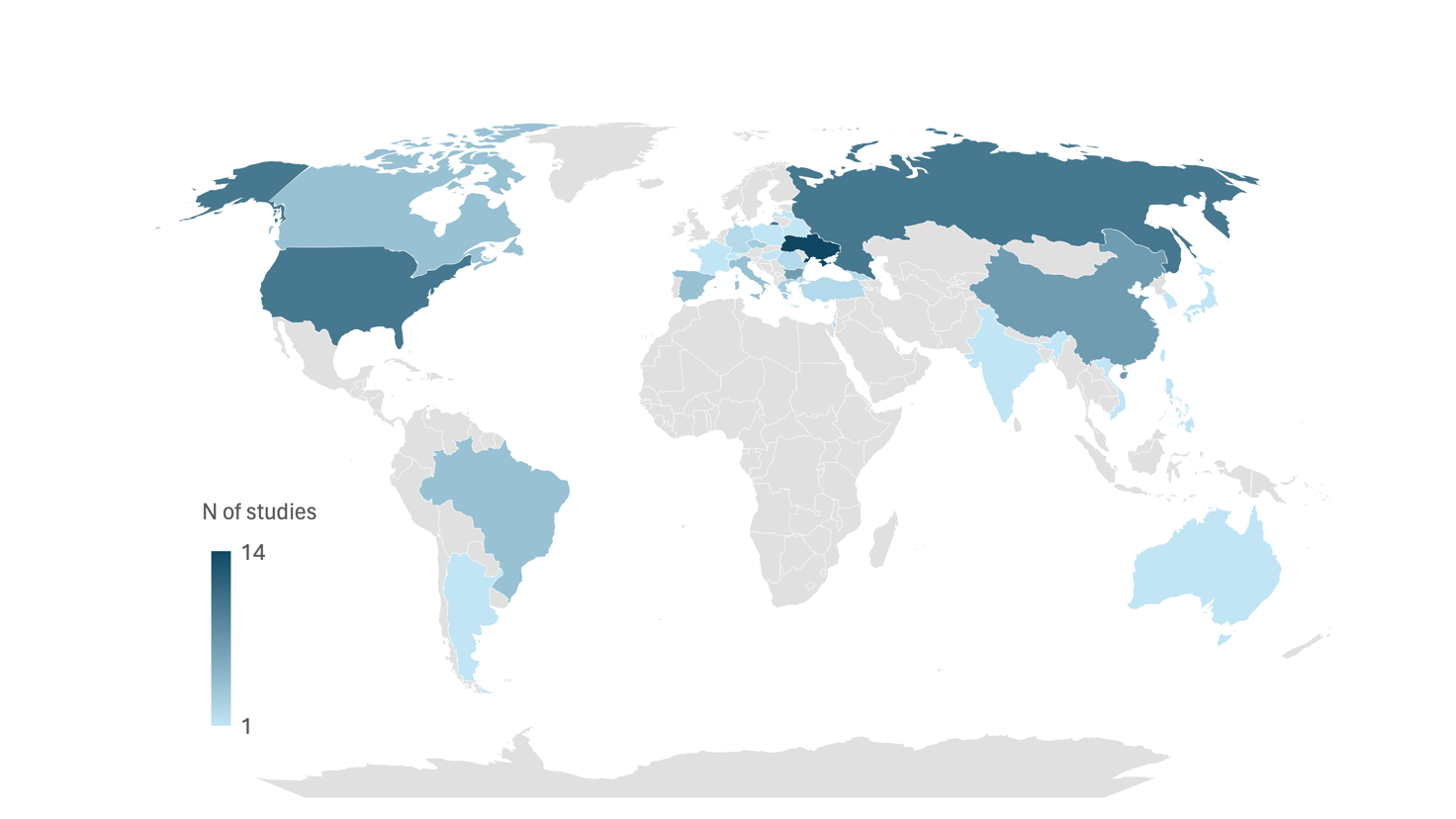


**Figure S1.** World map with countries colored according to the number of times they were listed among the top three countries with regards to participant enrollment.

ALT TEXT: World map with countries colored according to the number of times they were listed among the top three countries with regards to participant enrollment.
